# Targeting Autophagy for Oncolytic Immunotherapy

**DOI:** 10.3390/biomedicines5010005

**Published:** 2017-01-11

**Authors:** Lulu Hu, Ke Jiang, Chan Ding, Songshu Meng

**Affiliations:** 1 Institute of Cancer Stem Cell, Dalian Medical University Cancer Center, 9Lvshun Road South, Dalian 116044, China; hululucsc@163.com (L.H.); jiangke.3@163.com (K.J.); 2 Department of Avian Infectious Diseases, Shanghai Veterinary Research Institute, Chinese Academy of Agricultural Sciences, Shanghai 200000, China

**Keywords:** oncolytic virus (OV), autophagy, immunotherapy, cancer

## Abstract

Oncolytic viruses (OVs) are capable of exerting anti-cancer effects by a variety of mechanisms, including immune-mediated tumor cell death, highlighting their potential use in immunotherapy. Several adaptation mechanisms such as autophagy contribute to OV-mediated anti-tumor properties. Autophagy regulates immunogenic signaling during cancer therapy which can be utilized to design therapeutic combinations using approaches that either induce or block autophagy to potentiate the therapeutic efficacy of OVs. In this article, we review the complicated interplay between autophagy, cancer, immunity, and OV, summarize recent progress in the contribution of OV-perturbed autophagy to oncolytic immunity, and discuss the challenges in targeting autophagy to enhance oncolytic immunotherapy.

## 1. Introduction

Oncolytic viruses (OVs) are naturally occurring or engineered viruses that selectively infect and replicate in cancer cells and cancer-associated endothelial cells, triggering direct oncolysis. Recent studies reveal that OVs induce immunogenic cancer cell death (ICD), which includes immunogenic apoptosis, necrosis/necroptosis, pyroptosis, and autophagic cell death, upregulating the presentation or release of tumor-associated antigens (TAAs), damage-associated molecular patterns (DAMPs), and pathogen-associated molecular patterns (PAMPs) to the tumor microenvironment. These in turn activate antigen-presenting cells (APCs) and T cells to stimulate the adaptive immune response [1,2,3,4,5,6,7,8,9]. As such, OVs are now recognized as novel immunotherapeutic agents and, as such, OV-mediated cancer therapy has more recently been termed “oncolytic immunotherapy” [10,11].

Macroautophagy (hereafter referred to as autophagy), a cellular waste disposal process, is important for maintaining homeostasis. Autophagic dysfunction has been associated with various human diseases, such as cancer, neurodegenerative, and autoimmune disorders. Accumulating evidence demonstrates that autophagy, as a conserved defense mechanism, contributes to both innate and adaptive immune responses against microbial infections [12,13,14]. OVs have been shown to interfere with the cellular autophagic machinery either in cancer cells or in the tumor microenviroment for their replication and survival [15,16]. In addition to their involvement in anti-viral immunity, autophagy plays a dual role in cancer. Autophagy can act as a tumor suppressor mechanism or promote tumorigenesis in different cancer models. Although the complex interplay between autophagy and cancer has been extensively reviewed and discussed [17], it is worth noting that cellular autophagy that is induced by viral infection may enhance cross-presentation and processing of tumor antigens by dendritic cells (DCs) to activate T cells [18,19], thereby inducing anti-tumor immunity [20]. Currently, several clinical trials are ongoing to assess the effect of manipulating the autophagic process in the treatment of human cancers [21].

As an emerging new modality for the treatment of cancer, preclinical and clinical progress in the field of oncolytic immunotherapy has been extensively reviewed elsewhere [7,8,11,22,23,24,25,26,27,28]. In this review, we will focus on OV-regulated autophagy and other current strategies to target autophagy as novel approaches to enhancing oncolytic immunotherapy.

## 2. Autophagy, Immunity, and Cancer

### 2.1. Autophagy Machinery

The main features and markers of autophagy comprise of autophagosomes (double membrane cytoplasmic vacuoles) that can fuse with endosomes to form the amphisome. These vesicles then fuse with lysosomes to degrade the autophagic cargo [29]. The execution of autophagy involves more than 30 essential autophagy-related (*Atg*) genes, most of which are highly conserved from yeast to mammal [30]. These *Atg* gene products participate in four distinct stages of autophagosome biogenesis: initiation with Atg1 (in yeast) or its equivalent Ulk1 (in mammals) complex, vesicle nucleation with the Atg6 (Beclin-1 in mammals)-Class III phosphatidylinositol 3-kinase (PI3K) (hVps34) complex, vesicle elongation with the microtubule-associated protein light chain 3(LC3) lipidation, and vesicle fusion with the SQSTM1 complex. The major control complex for autophagy is mTOR (mammalian target of rapamycin), an evolutionarily conserved serine/threonine kinase, which inhibits initiation of the autophagy pathway when activated. Pivotal activators of mTOR include the Class I PI3K/Akt pathway, high Adenosine monophosphate/Adenosine Triphosphate (AMP/ATP) ratios, and several anti-apoptotic proteins such as Bcl-2 and Bcl-Xl [31,32,33]. In contrast to Class I PI3K, the Class III PI3K, with its subunit hVps34 forming a multi-protein complex that contains Beclin-1, positively regulates autophagic induction. Detailed information on autophagic induction and its regulation has been well-documented [34].

### 2.2. Autophagy and Cancer

Accumulating evidence indicates a context-dependent role of autophagy in cancer. Autophagy may function as a tumor-suppressive mechanism during early tumorigenesis, but its role in advanced cancer remains unclear. Direct evidence showing the tumor suppressor function of autophagy comes from the fact that certain ATG-proteins, such as Beclin-1, exhibit an anti-oncogenic function. Inactivation of autophagy-related genes, such as *Beclin-1*, leads to increased tumorigenesis in mice while overexpression of these genes (*Beclin-1*, *Atg5*) inhibit the formation of human breast tumors in mouse models [35]. The tumor suppressor function of Beclin-1 is supported by the genetic evidence that *Beclin-1* is monoallelically deleted in breast, ovarian, and prostate tumors [36,37].

Distinct to the role of autophagy in tumorigenesis, it is widely accepted that autophagy is required for the survival of established cancers [38,39]. In this regard, autophagy inhibitors could be useful as cancer therapeutics [40]. However, regression of tumor xenografts derived from a large number of human cancer cell lines is not detected upon inhibition of autophagy [41]. Although the autophagy inhibitor chloroquine (CQ) suppressed growth of cancer cell lines, whether its effect is autophagy-dependent remains elusive. Taken together, whether autophagy should be inhibited or activated remains controversial.

### 2.3. Autophagy, Immunity, and Cancer Immunotherapy

The interplay between autophagy and immunity has been widely recognized. On the one hand, autophagy can act as an important regulator of the cell immune response, where, for example, autophagy promotes the maturation of T cells and the survival of activated cytotoxic T-lymphocytes (CTL) [14,23,42,43]. However, the mechanism by which autophagy modulates such immune responses remains largely unknown. On the other hand, immune-inflammatory signals can also regulate autophagy [44], although the major common physiological activator of autophagy is starvation and growth factor deprivation [45]. The interaction between autophagy and cellular immunity has been extensively reported in the literature [46,47,48].

Of note, autophagy has been recently linked to cancer immunotherapy [43,49]. Autophagy potentiates the processing and presentation of tumor antigens and is crucial for mounting antigen-specific T cell responses [50], thereby stimulating anti-tumor immunity. Interestingly, cancer cells can escape immune surveillance by inhibiting autophagy. In this regard, autophagy enhancers may increase the efficacy of cancer immunotherapy. Overall, targeting autophagy-dependent anti-tumor immune responses may be a promising therapeutic approach for cancer treatment although the mechanisms underlying immunogenic potential of autophagy have not been well defined. It should, however, be noted that, although it has been appreciated that pharmacological modulation of autophagy alters the anti-tumor immune response, the associated clinical data are not presently available and await results from ongoing clinical trials combining autophagy modulators with chemotherapeutic agents (https://clinicaltrials.gov/ct2/show/NCT01438177?term=Chloroquine+and+cancer&rank=13https://clinicaltrials.gov/ct2/show/NCT00411788?term=rapamycin+and+cancer&rank=33https://clinicaltrials.gov/ct2/show/NCT00935961?term=rapamycin+and+cancer&rank=10).

## 3. Oncolytic Viruses (OV)-Modulated Autophagy in Oncolytic Immunotherapy

There is accumulating evidence to suggest that OVs perturb the cellular autophagy system in order to exert their anti-tumor activity. Ito et al. first showed that autophagy acts as a cell death mechanism upon OV treatment in glioma cells infected with conditionally replicating adenovirus [51]. Not surprisingly, subsequent studies indicated that many OVs either subvert or hijack the host autophagic machinery to enhance their own replication and anti-tumor activity. The interplay between OVs and autophagy has been discussed widely [16,52]. Recently, OVs have been reported to induce autophagy-dependent release of DAMP, including ATP, HMGB1, uric acid, and other immune-related molecules as well as tumor antigens by cancer cells [43], indicating that autophagy may contribute to OV-mediated immunotherapy. Indeed, several OVs have been shown to modulate autophagy to induce both innate and adaptive immune responses by contributing to antigen presentation and cytokine secretion during the oncolytic processes. A summary of the recent advances in OV-mediated autophagy in oncolytic immunotherapy is shown (Table 1).

Based on the reported contributions of OV-modulated autophagy to anti-tumor immune responses shown in Table 1, several conclusions can be drawn from these: (1) Most of the OVs promote the anti-tumor immune response by enhancing autophagy while few inhibit autophagy [61]. (2) The underlying mechanisms by which autophagy plays a role in oncolytic immunotherapy are largely unknown. (3) Most of the studies examining the effects of OV-modulated autophagy on the cellular immune response are performed in vitro using established tumor cell lines or in vivo mice models, but few studies have been carried out in clinical trials. (4) Data based on in vivo modulation of autophagy using combination strategies and their effects on the immune system have not been convincing.

## 4. Combination Strategies Using Oncolytic Virus and Chemical Agents for Stimulating and Inhibiting Autophagy

OVs have been used in combination with conventional agents that specifically target cancer cell signaling pathways [62,63,64,65,66,67,68]. Recently, combining OVs with existing immunotherapies such as immuno-oncology agents targeting different checkpoint pathways have been investigated in both preclinical studies and clinical trials [7,8,25,26,69]. Given that OV infections can interplay with the cellular autophagy machinery, the use of OVs in combination with autophagy modulators may augment anti-tumor immune responses, thereby delivering enhanced efficacy and providing greater clinical benefit to cancer patients. Pharmacological autophagy modulators may be used to enhance oncolytic immunotherapy by (1) inducing innate and adaptive immune responses by contributing to antigen presentation and cytokine secretion and (2) breaking immune tolerance in the tumor microenvironment and facilitating increased immune cell infiltration into the tumor [70]. A number of reviews have reported the potential anti-tumor effects of a variety of autophagy-modulating drugs in preclinical and clinical trials [38,39]. Among these drugs, RAD001 and hydroxychloroquine (HCQ), two well-known autophagy regulators, were shown to be thoroughly investigated in Phase I and Phase II trials in various types of cancer [38,39].

RAD001 is derived from rapamycin (sirolimus), a naturally occurring allosteric mTOR inhibitor [71]. RAD001 selectively targets mTOR to stimulate autophagy. In several preclinical trials, rapamycin or RAD001 has been used to increase the potency of various OVs, such as Ad OBP-405 in glioblastoma cells [72], Ad Δ-24-RGD and Ad ICOVIR-5 in glioma xenografts [73], HSV in human breast, cervical, and esophageal cancer cell lines [74], and vaccinia virus strains vvDD-EGFP and JX-594 in rats bearing malignant glioma (MG) [75]. However, combinations with RAD001 or other analogs of rapamycin and OVs have not yet been investigated in clinical trials. Of note, like most autophagy enhancers, rapamycin and its analogs do not selectively target autophagy and may ultimately result in off-target effects.

HCQ (hydroxychloroquine) is a derivative of CQ (Chloroquine) that interferes with lysosomal acidification, thereby blocking the late stages of the autophagic process. HCQ is currently being investigated in Phase I and Phase II trials in patients with various tumors [76]. Unfortunately, the concentrations of HCQ required to fully inhibit autophagy are not consistently achievable in the clinical setting, thus hindering the utilization of HCQ as an autophagy inhibitor to enhance OV-mediated anti-tumor effects. However, CQ has been used in combination with various OVs to enhance OV-mediated cytotoxicity in preclinical studies. Examples for this combination strategy include Ad dl922-947 in ovarian cancer IGROV1 cells [77] and in glioma U87MG cell-derived tumor xenografts [78] as well as NDV in cisplatin-resistant lung cancer cells [59], where combinations with CQ enhance OV-mediated oncolytic effects. It should be noted that whether CQ enhances OV-mediated oncolysis via inhibition of autophagy warrants further investigation as this has not, as yet, been full elucidated in preclinical and clinical trials, given that autophagy is not the only target of CQ or HCQ.

Given that both rapamycin and CQ play an important role in immunogenic signaling in cancer therapy, it is anticipated that such agents may offer potential in augmenting anti-tumor immune responses in OV-based clinical trials. To date, although few reports show that autophagy modulators can enhance OV-mediated anti-tumor immune responses, recent studies by Klein et al. indicate that, during the adenoviruses-mediated oncolytic process, viral antigens are processed by JNK-mediated autophagy and that autophagy was required for their presentation [55]. Based on a subsequent immuno-oncology study, the authors suggest that a combination of adenoviruses with autophagy inducers may enhance the processing and presentation of cancer-specific antigens incorporated into capsid proteins [55].

In addition to the use of pharmacological modulators of autophagy, targeting autophagy to enhance oncolytic immunotherapy can be achieved by engineering OVs to express autophagy-inducing genes, such as Beclin-1 [78] and regulators of the mTOR pathway [51,79]. This approach, in particular, may be useful for treating cancers that are resistant to apoptosis. Another obvious benefit from using this approach is that it may avoid potential side effects induced by autophagy-modulating drugs.

Of interest, a combination of two different kinds of OV that interact with autophagy machinery may also achieve unexpected enhanced anti-tumor immune responses given that different OVs utilize diverse strategies/mechanisms targeting autophagy and the immune system. In fact, it has been reported that using combined infections with OVs reovirus plus Newcastle disease virus, and reovirus plus parvovirus, exerts synergistic anti-tumor effects in U87MG cells in vitro and in vivo [80]. Whether autophagy is involved in this observed anti-tumor effect is not known. Nonetheless, this approach holds promise and warrants further investigation.

## 5. Future Perspectives

Advances in our understanding of the complex interplay between OVs, autophagy, and cancer, in addition to that of the immune system, have provided a foundation for the development of a potential combinatorial therapeutic approach using OVs and autophagy modulators. However, several hurdles need to overcome and addressed in more detail prior to the use of such combinations: (1) The mechanisms by which autophagy modulators regulate OV-induced anti-tumor immune responses are largely unknown. In particular, one must consider the potential negative impact of targeting tumor cell autophagy on immune cells. (2) The effect of targeting autophagy in the context of the tumor microenvironment must also be considered, as most studies have focused on the impact of autophagy modulation on tumor cells only. (3) The methods by which OVs and autophagy modulators are delivered in vivo and the timing and sequence of administration can significantly impact their efficacy. Thus, much work remains to be carried out to further refine this strategy and integrate these into routine clinical practice.

## Figures and Tables

**Table 1:** Summary of Oncolytic Virus (OV)-modulated autophagy in oncolytic immunotherapy.

Oncolytic Virus (OV)	Impact of OV on Autophagy	Impact of Autophagy on Oncolytic Immunotherapy	Key References
Herpes simplex virus HSV-2 (ΔPK)	Modulates the tumor microenvironment through autophagy-dependent pathways.	Decreased tumor cell secretion of the type 2 immunosuppressive and pro-cancerous cytokines, IL-10, and IL-18 and concomitant increased secretion of the pro-inflammatory cytokines TNF-α, GM-CSF, IL-6, and IL-1β. Upregulates the NKG2D ligand, MICA, expressed by cytotoxic NK and T cells, and downregulates the negative immune checkpoint regulator cytotoxic T-lymphocyte antigen-4 (CTLA-4)	[53]
Herpes simplex virus type 2 (ΔPK)	Induces autophagy by upregulating inflammatory cytokines through autophagy-dependent activation of TLR-2 signaling.	Upregulates the secretion of inflammatory cytokines TNF-α, granulocyte macrophage colony-stimulating factor and IL-1β through autophagy-mediated activation of Toll-like receptor 2 pathways	[54]
Ad(Δ24FvIII)	Induces autophagy through JNK activation.	Autophagy inducers may enhance the processing and presentation of cancer-specific antigens	[55]
Ad (5/3-D24-GMCSF)	Increases tumor cell autophagy.	Releases HMGB1 into serum in patients and elicits anti-tumor immune responses.	[56]
Ad (OBP-301)	Induces autophagy-associated cell death.	Produces the endogenous danger signaling molecule, uric acid which stimulates DCs to produce interferon-γ (IFN-γ) and interleukin 12 (IL-12).	[57]
Newcastle disease virus (NDV)	Induces autophagy in ICD.	Primes adaptive anti-tumor immunity.	[58]
NDV/FMW	Pharmacological modulation of autophagy; enhances the oncolytic effects of NDV/FMW.	Releases HMGB1.	[59]
Measles virus (MV-Edm)	Triggers SQSTM1/p62-mediated mitophagy.	Mitigates DDX58/RIG-I-like receptor signaling and the innate immune response.	[60]
